# The Effect of the Finishing Deformation Temperature on the Microstructure of CrVNb Micro-Alloyed Steel

**DOI:** 10.3390/ma18143234

**Published:** 2025-07-09

**Authors:** Gholam Ali Baqeri, Chris Killmore, Lachlan Smillie, Elena Pereloma

**Affiliations:** 1 School of Mechanical, Materials, Mechatronic and Biomedical Engineering, University of Wollongong, Wollongong, NSW 2522, Australia; 2 ARC Research Hub for Australian Steel Innovation, University of Wollongong, Wollongong, NSW 2522, Australia; chris.killmore@bluescopesteel.com; 3 BlueScope Steel Limited, Five Islands Rd, Port Kembla, NSW 2505, Australia; 4 Electron Microscopy Centre, University of Wollongong, Wollongong, NSW 2522, Australia; lsmillie@uow.edu.au

**Keywords:** thermomechanical processing (TMP), electron microscopy, ferritic micro-alloyed steel, interphase precipitation (IP)

## Abstract

This study explored the effects of the finishing deformation temperature on the microstructure and properties of CrVNb micro-alloyed steel following thermomechanical processing (TMP). The investigation encompassed the influence of the deformation temperature on the ferrite grain size, precipitate characteristics, hardness and flow stress. The microstructure characterization was performed using optical and electron microscopy techniques. The results show that decreasing the deformation temperature refined the ferrite grains, though a bimodal ferrite grain structure formed when the deformation temperature fell to about 100 °C below the Ar3 temperature. Additionally, lower deformation temperatures increased the number density of strain-induced precipitates (SIPs), whereas the density of finer precipitates (random and interphase precipitates (IPs)) decreased. The highest hardness was observed in a sample deformed at 950–850 °C temperatures. These findings highlight the impact of the finishing deformation temperatures on the microstructural and mechanical properties, providing valuable insights for optimizing steel processing conditions.

## 1. Introduction

High-strength low-alloy (HSLA) steels are an important class of materials renowned for their mechanical properties and wide industrial applications. These materials are notable for their good strength-to-weight ratio, toughness, formability, weldability and corrosion resistance, making them highly suitable for applications like automotive industry, oil and gas extraction, and construction [1,2,3]. The addition of small amounts of alloying elements, such as Nb, V, Cr, Ti and Mo, enhances the strength of the ferritic (α) matrix through three main mechanisms: grain size refinement, precipitation hardening and solid solution strengthening [4,5,6].

In addition to the chemistry of steel, the microstructure of these steels plays a pivotal role in determining their mechanical strength, toughness and overall performance [2,7,8,9]. There are different parameters used during thermo-mechanical processing that affect the properties of the final product, like the reheating temperature and time [6], cooling rate [10], amount of deformation [11] and its temperature, coiling temperature [12] and coiling time [13]. Amongst these, the deformation temperature stands out as one of the critical parameters.

The restoration mechanisms of austenite (γ) and their temperatures influence the microstructure of steel after TMP. To achieve an optimal combination of strength and toughness, it is important to understand the effect of deformation temperatures during hot rolling on the grain structure of austenite (γ). Tanaka et al. [14] divided the deformation during TMP into three different stages based on the temperature: (1) deformation in the recrystallization region that refines the final ferrite grain size through the recrystallization of austenite, (2) deformation in the non-recrystallization γ region that increases the grain boundaries and deformation bands density, and (3) deformation in the α–γ two-phase region that deforms the ferrite grains formed previously and produces the sub-structure in ferrite grains. However, Fukuda et al. [15,16] only considered deformation in austenite. Based on this category, deformation can be performed in the recrystallization region and/or non-recrystallization region. Deformation in the recrystallization region itself is divided into two types: type A represents the deformation at higher temperatures that does not contribute to the austenite refinement, while type B, which deforms at lower temperatures in the recrystallization region, leads to austenite grain size refinement. Panirgahi [17] classified the TMP based on the deformation temperature under five different regions: deformation in the recrystallized austenite region; in the non-recrystallized region; on the border of recrystallized and non-recrystallized regions; in the austenite–ferrite region; and finally, in the ferrite region.

Finishing deformation boosts the density of lattice defects, like grain boundaries, dislocations, shear bands and deformation bands [17]. It has been reported that these defects are potential ferrite nucleation sites [17,18,19]. Deformation in the non-recrystallization region causes the formation of an austenite pancaked structure, as the recrystallization of austenite cannot happen. Also, based on the theory of kinking suggested by Frank and Stroh [20], deformation bands nucleate on available inhomogeneities and extend if the applied stress reaches a critical value. Panirgahi et al. [17] reported the formation of deformation bands and twins in micro-alloyed steels when the reduction per pass is above 20%. Inagaki [18] believed that the deformation in the non-recrystallization region causes a local deformation in the region near the annealing twins, as well as austenite grain boundaries. Consequently, ferrite nuclei form in these regions during the austenite to ferrite transformation. This process leads to final ferrite grain size refinement.

The reduction in the finishing deformation temperature increases the density of these defects, which raises the stored energy in the material [21]. However, if the amount of this stored energy reaches the critical value, softening mechanisms, like DIFT, will take place, even at temperatures above A_r3_ [22,23,24,25].

In this research, we explored the relationship between finishing deformation temperatures during simulated hot strip rolling and the microstructural evolution of recently developed CrVNb micro-alloyed steel. By examining the mechanisms through which temperature alters the microstructure, we aimed to provide insights into optimizing the processing conditions for achieving the desired material properties.

## 2. Materials and Methods

The composition of the micro-alloyed steel used in this study was 0.11% C, 1.39% Mn, 0.26% Si, 0.03% Al, 0.011% Ni, 0.018% Cu, 0.002% Mo, 0.0129% N, 0.01% P, 0.004% S and a total percentage of microalloying elements (Cr-V-Nb) equal to 0.723 (wt.%). The as-cast steel was homogenized at 1300 °C for 30 h and then forged from 75 to 28 mm in thickness. The next step was the machining of specimens with 11 × 15 × 20 mm^3^ dimensions for TMP simulations using a Gleeble 3500 Thermal-Mechanical Simulator (Dynamic Systems Inc., Poestenkill, NY, USA).

**Figure 1:**
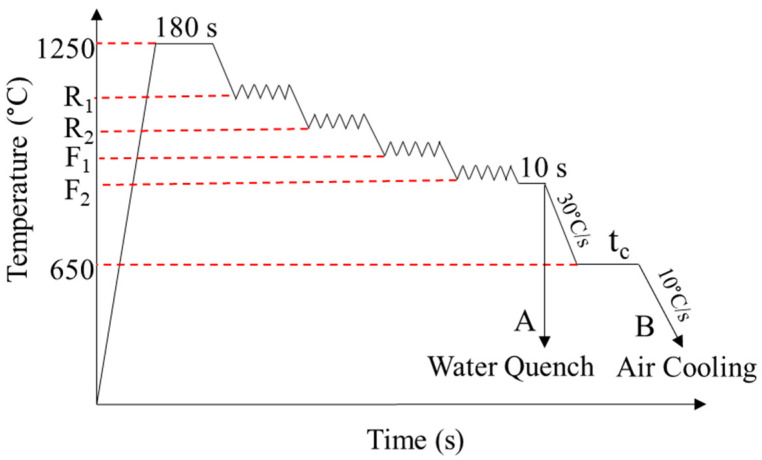
Schematic of TMP schedules of quenched and coiled samples after deformation at different temperatures (R and F represent roughing and finishing deformation temperatures, respectively, and t_c_ is the coiling time).

Figure 1 shows the schematic of the TMP simulation conducted in this study. The samples were reheated at 1250 °C for 180 s to ensure the complete dissolution of precipitates, particularly complex Nb(CN), which has been reported to dissolve at around 1150 °C [6]. The selected holding time also allowed for thermal field homogenization while minimizing the austenite grain growth. Following reheating, the samples underwent four plain strain compression steps above and below the non-recrystallization temperature (T_nr_ = 950 °C [26]). The thickness of the samples reduced from 11 to 2.2 mm after the deformation. It should be noted that the total strain in the non-recrystallization region in this study was 0.5 higher than that used in Ref. [26], which could influence the effective T_nr_.

The first two deformations, which were above T_nr_ (R_1_ and R_2_), represent the roughing rolling stage, and their temperatures were kept constants for all TMP schedules. The third and fourth deformations were performed at different temperatures (F_1_ and F_2_) below T_nr_ to study the effects of the finishing rolling temperatures on the microstructures of the specimens. A total applied strain of 1.5 was introduced during the TMP, which included four deformation passes (R_1_, R_2_, F_1_ and F_2_). Table 1 describes the finishing deformation temperatures for different schedules, as well as their microstructure characteristics. In this paper, each sample is labeled as “X-Y”, where X and Y represent the finishing deformation temperatures F_1_ and F_2_ (in °C), respectively (e.g., the “1000-900 sample” indicates finishing deformations at 1000°C and 900°C. After the last deformation stage, the samples were held at the deformation temperature for 10 s to promote thermal homogenization and reduce the localized microstructural effects that resulted from accumulated deformation below T_nr_. Although the temperature was below T_nr_, this short isothermal hold allowed for partial recovery, thereby reducing heterogeneities associated with prior deformation. For each condition, a sample was water quenched to room temperature to study the effect of the deformation temperature on the austenite microstructure, while another sample was cooled to 650 °C (at a rate of 30 °C/s) and held for 3600 s, which were the simulated coiling temperature and time, respectively. Then, the samples were slowly cooled down to room temperature (at a rate of 10 °C/s). It should be noted that all deformation steps were performed at the constant strain rate of 5 s^−1^.

To analyze the microstructure of specimens after the TMP, the samples that experienced air cooling (route B in Figure 1) were etched using 2% nital solution for about 10 s. The quenched samples were etched using 150 mL of distilled water, 4.5 g of picric acid, 1 mL Teepol as a wetting agent and 8 drops of HCl acid at 80 °C for 35 s [27]. Also, the quenched samples were etched again using 2% nital solution to reveal the ferrite formed during the deformation. The microstructure characterization was conducted using a Leica DMRM (Leica Microsystems, Wetzlar, Germany) optical microscope (OM) and FEI Helios NanoLab G3 CX (Thermo Fisher Scientific, Hillsboro, OR, USA) focused ion beam (FIB) field emission scanning electron microscope FEGSEM equipped with an Oxford instruments X-max^N^ 150 mm^2^ 123 eV SDD energy dispersive X-ray detector (Oxford Instruments, High Wycombe, UK). The FIB was operated at 5 kV and 11 nA in secondary electron (SE) mode.

To prepare the transmission electron microscope (TEM) lamellas using FIB, the Crystal Aligner computer program was used to determine the ideal geometry with the (110) plane normal to the observation direction from the Electron Backscatter Diffraction (EBSD) data [28]. The voltage and current of the initial trenching were 30 kV and 9.3 nA, respectively, while the final cleaning was performed at 30 kV and 2.5 nA. Carbon replicas of the samples were prepared to measure the number density of precipitates using TEM. First, the polished samples were etched for 10 s using 2% nital. A 15 nm thick carbon layer was then deposited on the surface using a Leica EM ACE600 Sputter Coater (Leica Microsystems, Wetzlar, Germany). After 24 h, the coated layers were scribed into 2 × 2 mm^2^ squares to facilitate the extraction of the carbon replicas. The samples were immersed in a solution that consisted of 2 vol.% HNO_3_, 5 vol.% ethanol and 93 vol.% distilled water, with slight agitation provided by a pipette during this step. Once the carbon replicas were detached, the solution was replaced with a rinsing solution of 5 vol.% ethanol and 95 vol.% distilled water. The rinsing process was repeated six times, after which the carbon replicas were collected on 400 square mesh copper grids.

A JEOL Cold FEGTEM (field emission gun TEM) JEM-F200 (JEOL Ltd., Tokyo, Japan) was used for the TEM analysis of the phases and precipitates at 200 kV. The high-resolution scanning transmission electron microscopy (STEM) investigation of precipitates was performed using a probe-corrected JEOL-ARM F-200 Cold FEGTEM (JEOL Ltd., Tokyo, Japan) at 200 kV. The energy-dispersive X-ray spectroscopy (EDS) analysis was performed using a JEOL Centurio SDD detector (JEOL Ltd., Tokyo, Japan) with a 100 mm^2^ detection area.

The quantitative analysis of the ferrite grain size and the area percentage of the secondary constituents was undertaken by ImageJ 1.48v freeware. About 1000 grains were measured for each condition and the average value was reported as the ferrite grain size. Vickers microhardness tests were performed using a MATSUZAWA Via-F microhardness tester machine (MATSUZAWA Co., Ltd., Akita, Japan). The average of 5 hardness measurements was considered as the microhardness for each sample.

## 3. Results

### 3.1. OM and Scanning Electron Microscopy (SEM)

**Figure 2:**
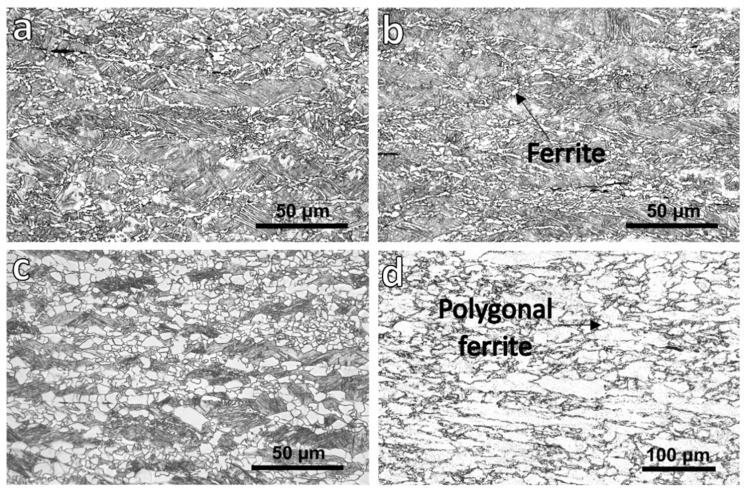
OM micrographs of samples after deformation, 10 s holding at the last deformation temperature, and water quenched with finishing deformation temperatures of (**a**) and (**d**) 1000–900, (**b**) 950–850 and (**c**) 900–780 °C. (**a**–**c**) show the samples etched by nital and (**d**) was etched by picric acid.

The OM micrographs of the quenched samples (experienced route A in Figure 1) are shown in Figure 2. The microstructures consisted of polygonal ferrite and martensite that was formed due to quenching. The ferrite grains mostly nucleated at the prior austenite grain boundaries and formed a necklace shape, which revealed the prior austenite grain structure. Sekine et al. [19] reported that when the etching of the austenite grain structure does not illustrate the microstructure, the austenite grain boundaries may be revealed from the situation of ferrite nuclei on an incompletely quenched sample. In Figure 2d (etched by solution with picric acid), the austenite displays a pancaked structure due to the deformation in the non-recrystallization region before the transformation. The prior austenite grain aspect ratio was measured from quenched samples etched using a solution that contained picric acid (see Section 2), which clearly revealed the austenite grain boundaries. A line–intercept method was applied on these micrographs, where lines were drawn parallel and perpendicular to the rolling direction. The average grain size in each direction was calculated by dividing the total line length by the number of intercepts, and the aspect ratio was obtained as the ratio of grain sizes in the two directions. The aspect ratio increased from 2.4 ± 0.4 to 3.5 ± 0.6, with a decrease in the finishing deformation temperatures from 1000–900 °C to 950–850 °C. However, there were some small polygonal ferrite grains within the austenite pancaked microstructure of samples with the deformation temperatures of 1000–900 and 950–850 °C (Figure 2d). Decreasing the finishing deformation temperatures from 1000–900 to 900–780 °C led to increases in the ferrite grains size and volume in the quenched samples (Figure 2a–c).

**Figure 3:**
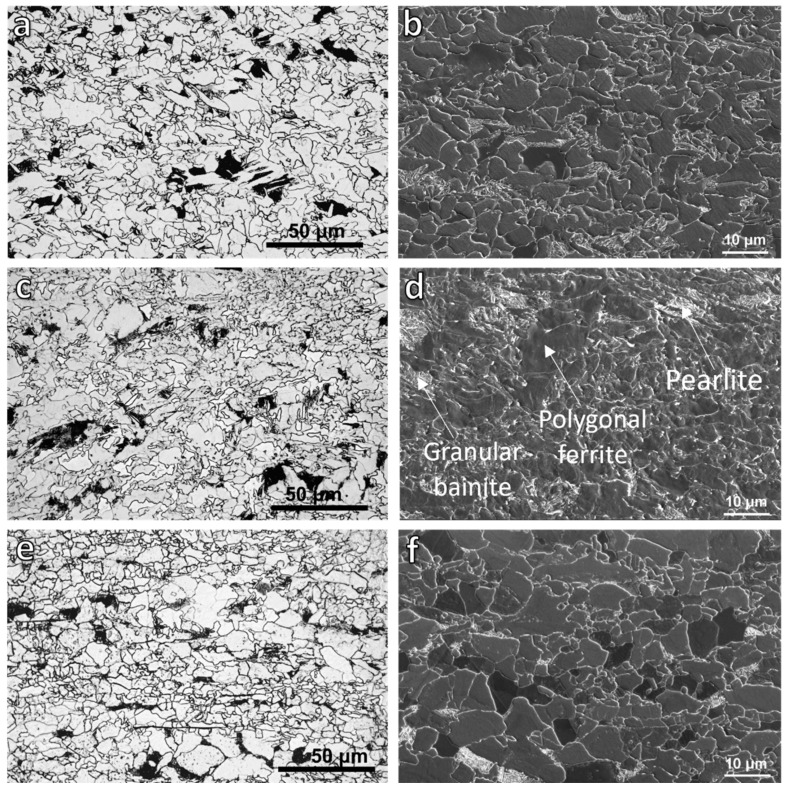
OM (**a**,**c**,**e**) and SEM (**b**,**d**,**f**) micrographs of samples with finishing deformation temperatures: (**a**,**b**) 1000–900, (**c**,**d**) 950–850 and (**e**,**f**) 900–780 °C, and after 60 min isothermal holding at 650 °C.

Figure 3 represents the OM and SEM micrographs of samples with three different deformation temperatures and coiled at 650 °C for 1 h (route B in Figure 1). The matrix consisted of polygonal ferrite, while pearlite and granular bainite—which were distinguished from each other based on their characteristic morphologies in higher magnification images—formed as secondary constituents. The contrast variation between ferrite grains in the SEM image, with some appearing bright and others dark, was attributed to differences in the crystallographic orientation: grains that have a [001] direction close to the normal to the plane of polish tend to etch less and appear brighter and bulged, while those with other orientations are more readily etched, and thus, appear darker [29].

The details of their microstructure characteristics are given in Table 1. The reduction in the finishing deformation temperatures from 1000–900 to 950–850 °C caused an average ferrite grain size refinement from 2.7 to 2.4 µm; however, further reduction of the deformation temperatures to 900–780 °C slightly increased the average ferrite grain size (2.5 µm) compared with the 950–850 °C deformation temperatures. Also, the lowest finishing deformation temperature led to the formation of a bimodal ferrite grain size, as can be seen from its OM and SEM images (Figure 3e,f). The area percentage of the second constituents for specimens with the deformation temperatures of 1000–900 and 950–850 °C were roughly about 13%, but the sample with the lowest deformation temperature had only 9% secondary constituents. The measurement of the pearlite interlamellar spacing of samples showed a considerable reduction in the pearlite lamellar spacing by decreasing the deformation temperatures to 900–780 °C. This can be attributed to the acceleration of austenite to ferrite and pearlite transformations that resulted from deformation at a lower temperature [30].

**Figure 4:**
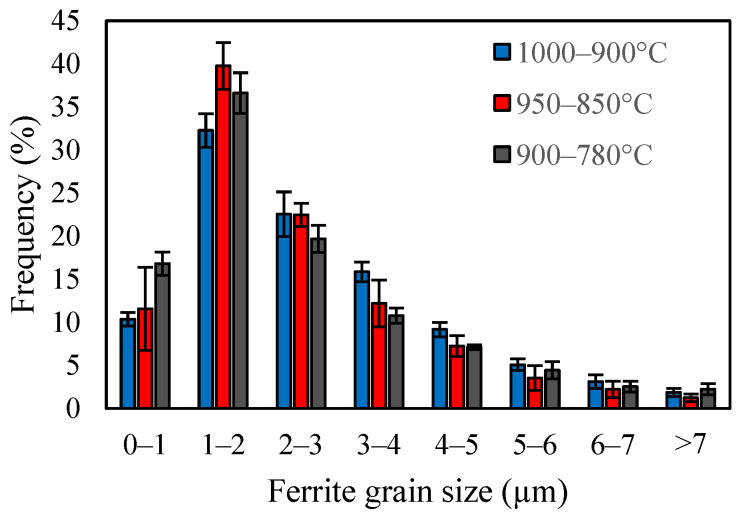
Ferrite grain size number distribution of samples with different finishing deformation temperatures.

The distribution of the ferrite grain size is presented in Figure 4. The decrease of deformation temperatures from 1000–900 °C to 950–850 °C caused a slight increase in the frequency of fine ferrite grains (smaller than 2 µm), while the number of grains larger than 3 µm decreased. The comparison of samples deformed at 950–850 °C and 900–780 °C showed that the lowest deformation temperature led to an increase in the number of fine grains (below 1 µm), as well as coarse grains (larger than 5 µm); however, the number of grains between 1 and 5 µm reduced. It should be noted that Figure 4 presents the number distribution, meaning that even a small change in the number of coarse grains can have a significant impact on the volume fraction of coarse grains.

The sample with the deformation temperatures of 950–850 °C had the highest Vickers hardness (294 HV), while the lowest deformation temperature led to the lowest Vickers hardness (257 HV) amongst these three deformation schedules.

### 3.2. Flow Stress Investigation

**Figure 5:**
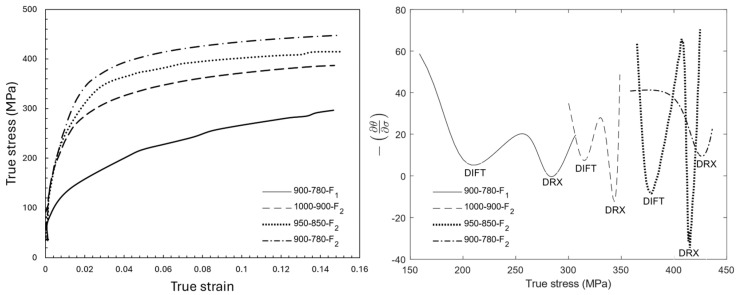
Plots of (**left**) true stress–strain curves and (**right**) −(∂θ/∂σ) for the samples with different finishing deformation temperatures. $\theta ={\left(\partial \sigma /\partial \epsilon \right)}_{\dot{\epsilon }}$.

The double-differentiation method was employed to study the softening mechanisms during the finishing deformation of samples. The details of this method have been described elsewhere [31,32,33,34,35]. Figure 5 illustrates the stress–strain curves and the variation in the second derivative of stress versus stress for different deformation temperatures. These curves had two minima, except for the deformation at 780 °C for the sample with the lowest deformation temperatures (900–780 °C), which showed only one. These minimum points show the onset stresses at which a softening mechanism activates. In each curve, the first minima is related to the activation of the deformation-induced ferrite transformation (DIFT), and the second one is where the dynamic recrystallization (DRX) of dynamically transformed ferrite starts to occur [35,36,37]. The deformation at 780 °C only activates the DRX of ferrite, as this temperature is considerably below A_r3_ (A_r3_ = 875°C [26]), where ferrite is thermodynamically stable. Given the softer nature of ferrite compared with austenite, most of the applied strain is stored in the ferrite, promoting the DRX of ferrite. Table 2 presents the critical strains and stresses for the activation of the DIFT and DRX at different deformation temperatures.

### 3.3. EDS Analysis of Precipitates

**Figure 6:**
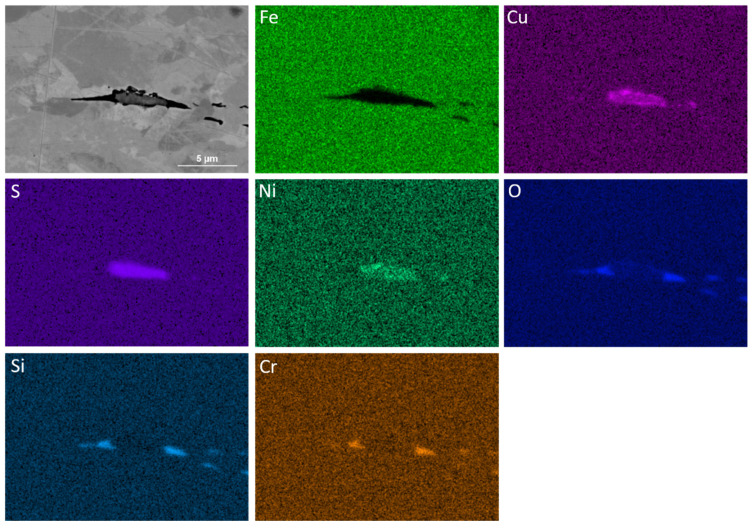
Backscattered electron (BSE) SEM image and EDS spectrum maps of a coarse inclusion in the sample with the finishing deformation temperatures of 1000–900 °C.

The EDS analysis of samples showed the formation of coarse sulfide and oxide inclusions, coarse iron carbide, medium sized copper sulfide and fine (Nb,V)C precipitates. Figure 6 illustrates a complex inclusion and the EDS map of its constituent elements. Its elongated morphology was due to the deformation of steel, which caused the stretching in the direction normal to the applied load. This complex inclusion consisted of (Cu,Ni)S, while chromium silicate was attached to the sides of the main body.

**Figure 7:**
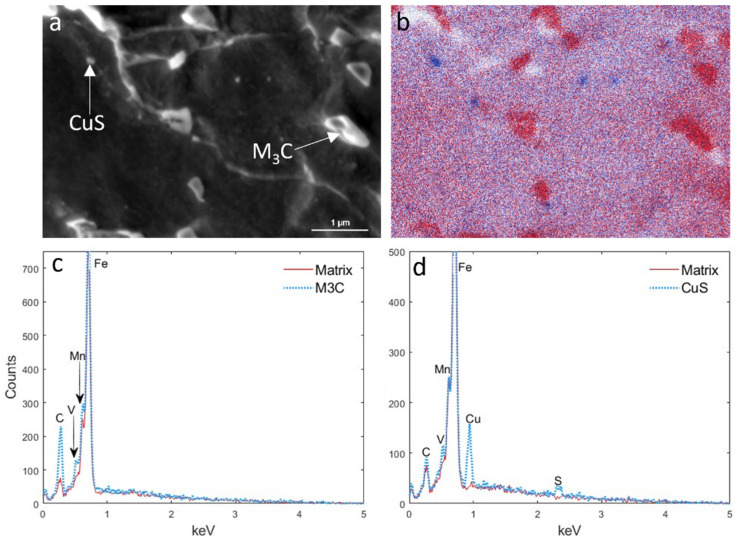
(**a**) SEM micrograph depicting coarse and medium-sized precipitates, (**b**) EDS maps of C (red) and Cu and S (blue) in the sample with deformation temperatures of 950–850 °C, and EDS spectra corresponding to the (**c**) iron carbide and (**d**) CuS precipitates shown in (**a**).

**Figure 8:**
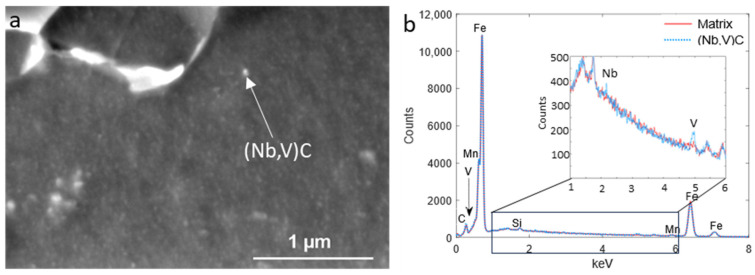
(**a**) SEM image showing a fine (V,Nb)C precipitate in the sample with finishing deformation temperatures of 950–850 °C and (**b**) corresponding EDS spectra of the pointed precipitate in (**a**) (the inset zooms in on the energy range of 1–6 keV, highlighting the Nb and V peaks).

The coarse M_3_C precipitates, where M consisted of Fe, Mn and V, were formed mostly at the ferrite grain boundaries; however, some formed within the grains (Figure 7a). The point EDS analysis of these particles demonstrated that the C, Mn and V peaks increased compared with the matrix (Figure 7c). Smaller precipitates, which formed mainly at the sub-grain boundaries, were identified as copper sulfide, as shown by the EDS maps of Cu and S (blue) in Figure 7b and the EDS spectrum in Figure 7d. Although there was no (Nb,V)C detectable in the EDS map because of their small size, the point EDS revealed their existence, as seen from Figure 8. It is well established that the partial substitution of Nb by V within (V,Nb)C precipitates reduces the lattice misfit between the precipitate and ferrite and lowers the overall Gibbs free energy, promoting the formation of such complex precipitates [38].

**Figure 9:**
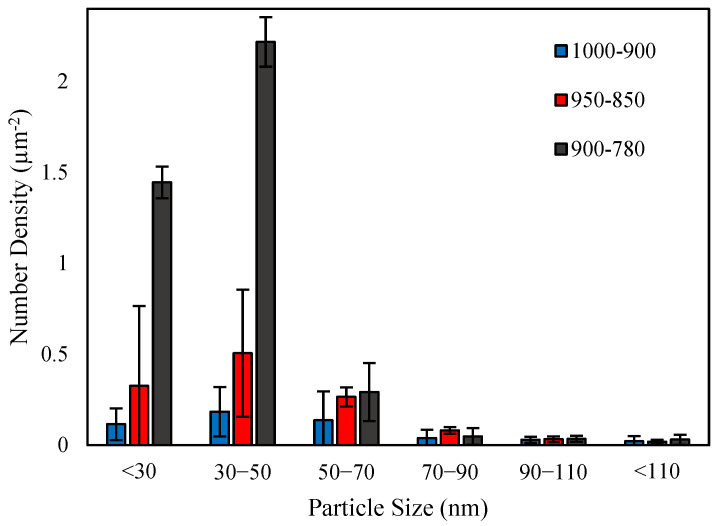
Size distribution of medium-sized precipitates of samples with different finishing deformation temperatures.

The number density distribution of precipitates is illustrated in Figure 9. It should be noted that this figure only shows the particles that are observable in SEM and are larger than ~20 nm. The total number of particles increased with decreasing deformation temperature (Table 1). The average size of the precipitates did not change considerably by decreasing the deformation temperatures from 1000–900 to 950–850 °C (about 58 nm). However, it decreased to 38 nm for the sample with the lowest deformation temperature. It can be seen from Figure 9 that the number density of particles smaller than 50 nm increased significantly in the sample with the deformation temperatures of 900–780 °C compared to the other two samples with higher deformation temperatures.

### 3.4. TEM Characterization

**Figure 10:**
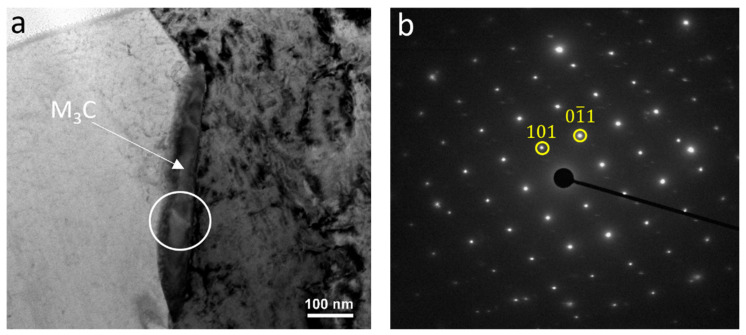
(**a**) Bright-field TEM micrograph representing the formation of a M_3_C particle along the ferrite grain boundary in the sample with the deformation temperatures of 900–780 °C and the (**b**) SADP from the circle in (**a**) (zone axis of cementite was $\bar{1}11$).

Figure 10a is the bright-field TEM micrograph of the sample deformed at 900–780 °C, showing the formation of medium-sized precipitates along the grain boundaries. The selected area diffraction pattern (SADP) taken from the encircled region in Figure 10a is displayed in Figure 10b, confirming that the grain boundary phase was cementite (M_3_C). The diffraction pattern corresponded to the orthorhombic crystal structure of cementite.

**Figure 11:**
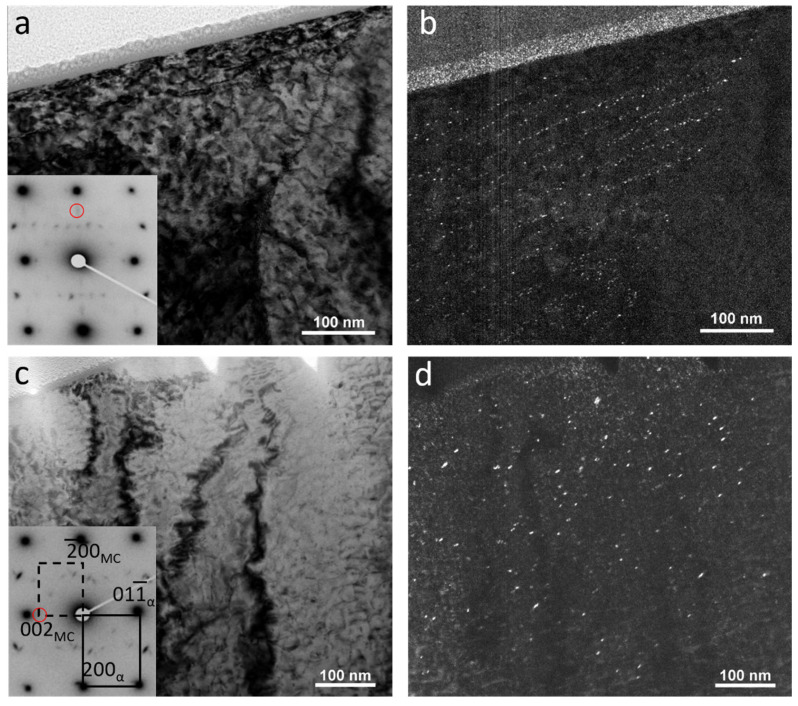
(**a**,**c**) Bright-field TEM micrographs and their corresponding diffraction patterns, (**b**,**d**) dark-field TEM micrographs illustrating the distribution of fine precipitates in steel with finishing deformation temperatures of (**a**,**b**) 1000–900 °C and (**c**,**d**) 900–780 °C. The zone axis was [011]_α_ and the red circles represent the diffraction spots corresponding to the dark-field images.

Figure 11 is the bright-field and dark-field images of samples with the highest and lowest deformation temperatures (1000–900 and 900–780 °C), as well as their related diffraction patterns. Small red circles in the diffraction patterns show the positions of diffraction spots that the dark-field images have been taken from. The precipitates maintained the Baker–Nutting (B-N) orientation relationship (OR) with ferrite, as represented by the following crystallographic relationships (Equation (1)) [39,40,41,42]:

(1) $${\left(001\right)}_{MC}\;//\;{\left(001\right)}_{\alpha },\;\;[{110]}_{MC}\;//\;{\left[100\right]}_{\alpha }$$

**Figure 12:**
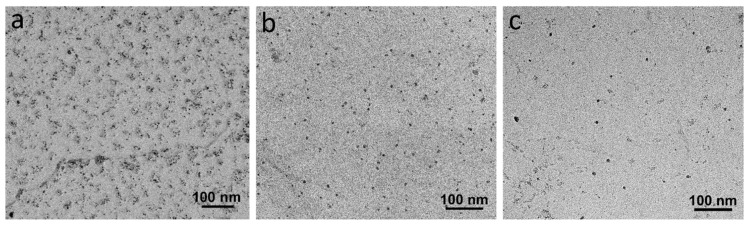
TEM micrographs obtained from the carbon replica samples with the deformation temperatures of (**a**) 1000–900 °C, (**b**) 950–850 °C and (**c**) 900–780 °C.

**Figure 13:**
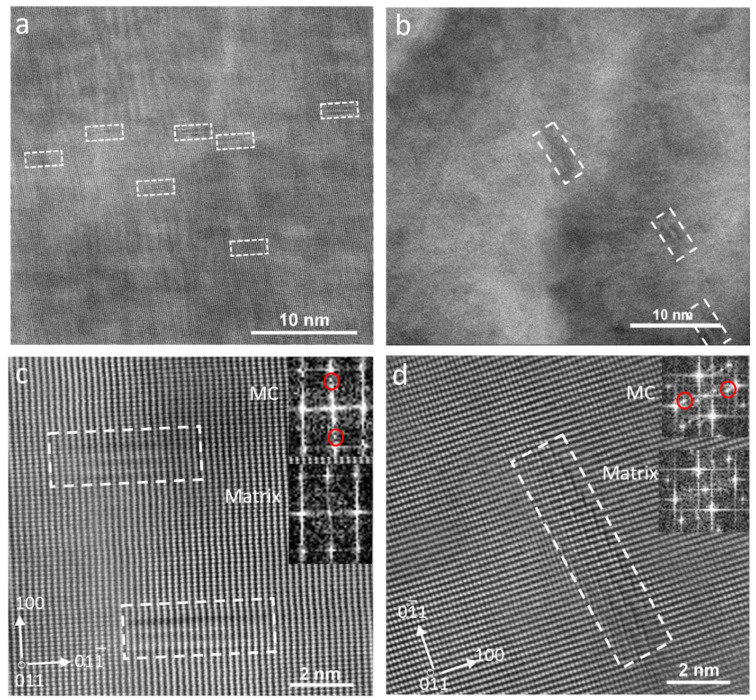
STEM HAADF micrographs illustrating fine (V,Cr,Nb)C precipitates after deformation, coiling and air cooling. The dashed white rectangles indicate the locations of the precipitates, and the red circles in the insets highlight characteristic diffraction spots corresponding to the (V,Cr,Nb)C phase. (**a**,**c**) 1000–900 °C and (**b**,**d**) 900–780 °C.

**Figure 14:**
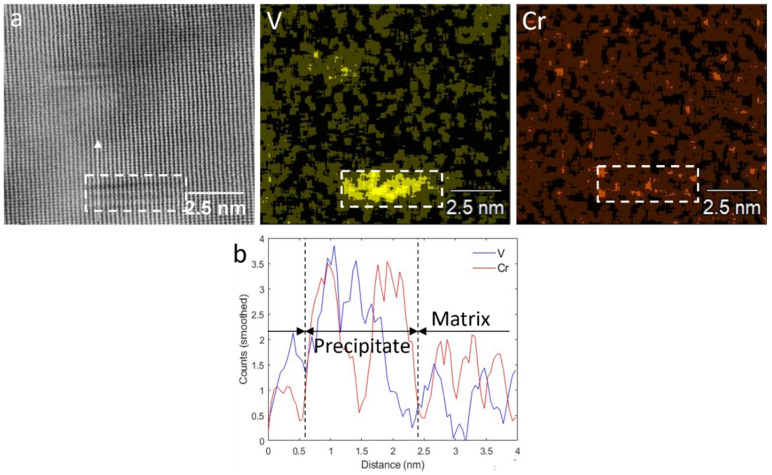
(**a**) STEM HAADF micrograph and EDS elemental maps (V and Cr) of precipitates shown in Figure 12c in the steel with a finishing deformation temperatures of 1000–900 °C; (**b**) linescan EDS corresponding to the white arrow in (**a**).

Although the rows of IPs are clearly distinguishable in the sample with the deformation temperatures of 1000–900 °C, precipitates seemed to form randomly in the sample with the lowest deformation temperature. Decreasing the deformation temperatures from 1000–900 °C to 900–780 °C caused an average fine precipitate size increase from 2.5 ± 0.8 to 4.9 ± 0.5 nm, while their number density reduced from 2546 ± 952 to 576 ± 169 µm^−2^ (Table 1). The TEM micrographs in Figure 12, which were obtained from carbon replica samples, illustrate the variations in the precipitate size and number density of the IP/random precipitates with decreasing deformation temperature. The high-angle annular dark-field (HAADF) images of the samples with the deformation temperatures of 1000–900 and 900–780 °C in Figure 13 clearly show the larger size of the precipitates in the sample with 900–780 °C deformation temperatures. As the last deformation in this sample was performed about 100 °C below the A_r3_ temperature, where a considerable amount of ferrite with precipitates had already formed, the interaction between these precipitates and dislocation movements during the deformation caused the formation of ledges at the precipitate/matrix interface (Figure 13d). Pereloma et al. [43] reported the same phenomena after the deformation of ferritic steel that contained VC IPs. The comparison of the Fast Fourier Transformations (FFTs) of MC (M = V, Cr and Nb) with the FFT of matrix (Figure 13c,d)) revealed diffraction spots of MC with the B-N OR relative to the matrix. The EDS analysis of precipitates illustrates the segregation of V atoms, while Cr atoms slightly accumulated around the V-rich core (Figure 14). However, the EDS did not show the presence of Nb, probably due to the small amount of Nb in the composition.

## 4. Discussion

The ferrite grains observed in the OM micrographs of the quenched sample deformed at 1000–900 °C (Figure 2a) as a result of the activation of the DIFT mechanism, as the A_r3_ temperature for this steel was measured as 875 °C in Ref. [24]. Since the deformation in this experiment was greater than in Ref. [24], the actual A_r3_ temperature was likely higher. This increase in A_r3_ suggests that the diffusional transformation of austenite to ferrite also could have occurred [44]. In the cases of the other two quenched samples (Figure 2b,c), the formed ferrite could be dynamically transformed ferrite and pro-eutectoid ferrite that formed during the 10 s holding after the deformation and before the quenching, as their last deformation temperatures were below A_r3_.

The plots of the second derivative of the stress–strain curves (Figure 5) confirmed the occurrence of the DIFT and DRX during the F2 deformations of the 1000–900 °C and 950–850 °C samples. However, these softening mechanisms arose during the F1 deformation (at 900 °C) of the sample with the lowest deformation temperatures. As the F1 deformation temperatures of the 900–780 °C sample was below T_nr_ (at 900 °C), the deformation caused an increase in the defect density, and consequently, in the stored energy. Thus, the accumulated strain was enough for the activation of the DIFT and DRX. Gosh et al. [45] showed that the mechanism of nucleation of a DIFT is displacive, while the growth mechanism is diffusional. Therefore, the time of growth influences the DIFT grain size. The DIFT grains in the sample with the deformation temperatures of 900–780 °C started to form during F1 at 900 °C, while in the other two samples, a DIFT was initiated during F2 in two other samples (Figure 5). The 900–780 °C sample experienced cooling from F1 to F2, deformation at F2 and 10 s of isothermal holding after F2. In contrast, the other samples began the DIFT only at F2, meaning the ferrite grains formed closer to the end of the process, and thus, had less time to grow before quenching. Therefore, the earlier initiation of the DIFT in the 900–780 °C sample resulted in more available time for grain growth compared with the other conditions. Also, the deformation at 780 °C led to the formation of more defects in this sample compared with those present in the samples deformed at 1000–900 and 950–850 °C because of more work hardening at lower temperature where the static restoration mechanisms were restricted [46]. A higher density of defects (especially the dislocation density) facilitated the diffusion of alloying elements and accelerated the growth of ferrite grains. This is why the volume fraction and grain size of ferrite were higher in the quenched sample with the deformation temperatures of 900–780 °C than in the 1000–900 and 950–850 °C ones (Figure 2).

The comparison of the critical strain for the activation of the DIFT and DRX (Table 2) shows that the reduction in the finishing deformation temperatures from 1000–900 °C to 950–850 °C led to less strain needed for the activation of the DIFT. Ferrite is thermodynamically unstable at temperatures above A_r3_ in an undeformed sample and increasing the temperature in this region increases the Gibbs free energy of ferrite. In other words, the activation of DIFT at higher temperatures demands more strain to overcome the larger thermodynamic barrier of transformation [35,36].

The refinement of the ferrite grain size at the deformation temperature of 950–850 than 1000–900 °C (Figure 3 and Table 1) can be attributed to the higher stored energy from plastic deformation at the lower temperatures (950–850 °C). This increased stored energy enhances the driving force for the austenite-to-ferrite transformation and reduces the activation barrier for ferrite nucleation, thereby promoting the formation of finer grains [39].

The formation of a large amount of DIFT grains during F1 in the sample deformed at 900–780 °C ended with a bimodal ferrite grain size after coiling (Figure 3e,f). The DIFT grains that formed at 900 °C could grow fast because of the facilitation of alloying elements diffusion. However, the new ferrite grains formed during cooling and isothermal holding at 650 °C could not grow at the same pace as the DIFT grains at higher temperatures. It is needless to mention that the DIFT grains also experienced more time to grow.

Another reason for the formation of the bimodal ferrite grain size in the sample with the lowest finishing deformation temperature was that as the last deformation step (at 780 °C) was performed about 100 °C below the A_r3_ temperature, both the deformed and undeformed ferrite grains coexisted during the isothermal holding at 650 °C. Undeformed ferrite grains formed either through the DRX of deformed ferrite during F2 (Figure 5) or during the cooling and coiling after this deformation step. These new undeformed ferrite grains that nucleated at the austenite/deformed ferrite interfaces could grow rapidly into the deformed ferrite. This rapid growth of undeformed ferrite into the deformed ones and the recovery of deformed ferrite led to the bimodal ferrite grain size after the isothermal holding. The reason for the growth of undeformed ferrite into the deformed ones was the difference in their stored energies and strain-induced boundary migration (SIBM) [47,48].

**Figure 15:**
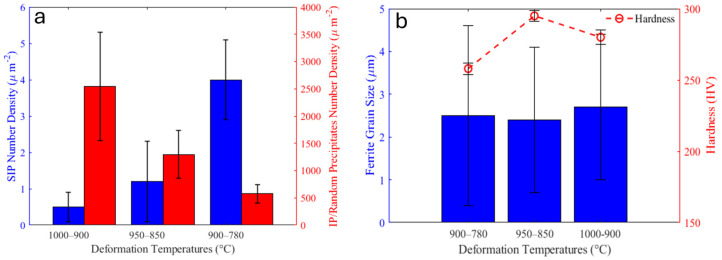
(**a**) Strain-induced and IPs/random precipitates number densities and (**b**) average ferrite grain size and hardness of samples with different finishing deformation temperatures (SIP and IP stand for strain-induced precipitate and interphase precipitate, respectively).

The reduction in the finishing deformation temperature also affected the number density and size of the SIPs. Increasing the number density of the SIPs after the deformation at lower temperatures of 950–850 °C compared with 1000–900 °C can be attributed to the higher work hardening and increasing the dislocations density and other defects, which are favorable nucleation sites for SIP [49]. However, their average size did not change considerably with this reduction in the deformation temperature. The considerable number density increment and average size refinement of the SIPs in the sample deformed at 780 °C also related to the formation of a dislocations network due to the deformation at a lower temperature, which promoted the nucleation and facilitated the diffusion of alloying elements through this network to the precipitate’s nuclei [49,50] (Figure 15a and Table 1).

Although SIPs control the austenite grain growth, they also consume the carbide-forming alloying elements that have the potential of forming small-sized precipitates during the austenite-to-ferrite transformation or in ferrite during coiling [51]. Interphase precipitates and random precipitates that form in ferrite are more efficient than SIPs in controlling the dislocation movements and precipitation strengthening. The formation of a higher number density of SIPs in the sample with the deformation temperatures of 900–780 °C left a lower amount of alloying elements dissolved in the austenite. With the reduction in the concentrations of Cr, V and Nb in the austenite, the driving force for the nucleation of precipitates during austenite to ferrite transformation and after transformation reduced, and consequently, the number density of IPs and random precipitates decreased (Figure 15a). On the other hand, in the sample deformed at 900–780 °C, the DIFT grains that had formed during F1 experienced deformation at 780 °C, which resulted in the formation of dislocations and deformation bands in them. These defects promoted the diffusion of alloying elements and caused the growth of precipitates. This was the reason for the larger sizes of random precipitates in the sample with the deformation temperatures of 900–780 °C than those of the IPs in the 1000–900 °C sample.

The comparison of the hardness of samples with different deformation temperatures revealed that the sample deformed at 950–850 °C had the highest hardness, while the lowest deformation temperatures (900–780 °C) showed the least hardness among these three schedules (Figure 15b and Table 1). It can be seen from Figure 15b that the refinement of ferrite grains in the 950–850 °C sample improved the hardness. It should be considered that the deformation at temperatures lower than 1000–900 °C increased the dislocation density, which assisted in improving the mechanical properties.

On the other hand, there were three reasons for the lower hardness of the 900–780 °C sample. First, the lower number density of random precipitates in this sample compared with the other two schedules (Figure 15a). Second, the bimodal ferrite grain size in this sample, which deteriorated the mechanical properties [52,53]. Finally, a lower percentage of secondary constituents in this sample than in the other two samples with higher deformation temperatures (Table 1). The reason for the lower amount of secondary constituents in this sample was the acceleration of the austenite-to-ferrite transformation due to the lower deformation temperatures and larger driving force for the transformation. In other words, the austenite-to-ferrite transformation started earlier and lasted longer than in the samples with higher deformation temperatures.

## 5. Conclusions

This investigation into the effect of finishing deformation temperatures on the microstructure of Cr-V-Nb micro-alloyed steel led to the following conclusions, which are firmly supported by the experimental results and detailed analyses presented in this study:

1. Reducing the finishing deformation temperatures to 950–850 °C refined the ferrite grains due to increased stored energy and acceleration of ferrite nucleation. At 900–780 °C, bimodal grains formed because the early DIFT grains grew fast at a high temperature, while the later-formed ferrite grew slower, and the strain-induced boundary migration during isothermal holding caused uneven grain growth.
2. The DIFT and DRX softening mechanisms occurred during the last finishing deformation of the 1000–900 °C and 950–850 °C schedules, while they took place during the third deformation stage in the sample with deformation temperatures of 900–780 °C because this stage was performed below T_nr_.
3. The finishing deformation schedule of 950–850 °C had a higher number density of SIPs than the 1000–900 °C schedule, while the average sizes were almost equivalent. This was due to the increased work hardening creating more nucleation sites. However, a higher number of SIPs with the smaller average size formed in the sample with the lowest finishing deformation temperatures.
4. The formation of large numbers of SIPs in the sample with the deformation schedule of 900–780 °C left a lower amount of dissolved alloying elements in the austenite, and subsequently, in the ferrite. This caused a reduction in the number density of random precipitates in this sample compared to the samples with higher deformation temperatures.
5. The 950–850 °C finishing deformation schedule resulted in the highest hardness between among the three schedules due to a finer ferrite grain size. The sample with the lowest deformation temperatures exhibited the lowest hardness because of the lower number density of fine precipitates, bimodal ferrite grain size and lower percentage of secondary constituents.

## Figures and Tables

**Table 1:** The characteristics of samples with different finishing deformation temperatures.

Finishing Deformation Temperatures (°C) (F_1_–F_2_)	1000–900	950–850	900–780
Ferrite average grain size (µm)	2.7 ± 1.7	2.4 ± 1.7	2.5 ± 2.1
Area percentage of the second constituents (%)	12.7 ± 0.4	13.1 ± 0.6	9.2 ± 0.4
Pearlite interlamellar spacing (nm)	179 ± 31	191 ± 29	110 ± 11
Pearlite colonies average size (µm)	4.3 ± 2.8	4.1 ± 2.3	2.4 ± 1.7
Strain-induced precipitates average size (nm)	58 ± 30	57 ± 29	38 ± 15
Strain-induced precipitates number density (µm^−2^)	0.5 ± 0.4	1.2 ± 1.1	4.0 ± 1.1
IPs/random precipitates average size (nm)	2.5 ± 0.8	3.9 ± 0.3	4.9 ± 0.5
IPs/random precipitates number density (µm^−2^)	2546 ± 952	1298 ± 444	576 ± 169
Hardness (HV)	279.9 ± 5.2	294.7 ± 3.8	257.7 ± 4.1

**Table 2:** Critical strains ($\varepsilon_{c})$ and stresses ($\sigma_{c})$ for the activation of the DIFT and DRX related to the curves shown in Figure 5.

Deformation Step	DIFT		DRX	
	$\varepsilon_{c}$	$\sigma_{c}$ (MPa)	$\varepsilon_{c}$	$\sigma_{c}$ (MPa)
1000-900-F_2_	0.09	315	0.15	343
950-850-F_2_	0.05	379	0.11	408
900-780-F_1_	0.07	208	0.14	284
900-780-F_2_	-	-	0.08	425

## Data Availability

The datasets presented in this article are not readily available because the data are part of an ongoing study and covered by intellectual property agreement with BlueScope Steel Ltd. Requests to access the datasets should be directed to Mr Gholam Ali Baqeri.
